# Pulmonary valve replacement after right ventricular outflow tract reconstruction with homograft vs Contegra®: a case control comparison of mortality and morbidity

**DOI:** 10.1186/s13019-018-0698-5

**Published:** 2018-01-17

**Authors:** Nicolas Poinot, Jean-Francois Fils, Hélène Demanet, Hugues Dessy, Dominique Biarent, Pierre Wauthy

**Affiliations:** 1 Department of Cardiac Surgery, Hôpital Universitaire des Enfants Reine Fabiola (HUDERF), Université Libre de Bruxelles, Avenue Jean Joseph Crocq 15, 1020 Brussels, Belgium; 2 Ars Statistica, Hôpital Universitaire des Enfants Reine Fabiola, Université Libre de Bruxelles, 1020 Brussels, Belgium; 3 Department of Cardiology, Hôpital Universitaire des Enfants Reine Fabiola, Université Libre de Bruxelles, 1020 Brussels, Belgium; 4 Intensive Care Unit, Hôpital Universitaire des Enfants Reine Fabiola, Université Libre de Bruxelles, 1020 Brussels, Belgium

**Keywords:** Contegra®, Congenital heart disease, Homograft, Allograft, Pulmonary valve, Redo surgery, Right ventricular outflow tract reconstruction

## Abstract

**Background:**

Repair of congenital heart defects involving the right ventricular outflow tract may require the implantation of a right ventricle to pulmonary artery conduit. This conduit is likely to be replaced during childhood. This study compares the operative outcomes of the replacement procedure of Contegra® and homografts in pulmonary position.

**Methods:**

From 1999 to 2016, 82 children underwent 87 right ventricle to pulmonary artery conduit replacements (60 Contegra® and 27 homografts). Demographics, operative and clinical data were obtained through a retrospective review of the medical records. The two groups were matched for comparison using propensity score matching. All the procedures were performed by the same team of surgeons.

**Results:**

No statistically significant difference was observed between the two groups when considering the operative data for anesthesia, surgery, cardiopulmonary bypass and aortic clamping durations. A peroperative complication rate of 13.47% and 15.36% in Contegra® and homograft replacement groups respectively (*p* value = 0.758) was observed. There was no difference regarding the blood loss and fluid input. No statistically significant difference was observed between the two groups for the post-operative morbidity. We considered the Pediatric Risk of Mortality (PRISM) score, the day of extubation, the day of withdrawal of inotropic drugs, the length of the intensive care unit stay and the length of hospital stay. The overall mortality is 2.3% but there is no statistically significant difference between the two groups.

**Conclusion:**

Right ventricle to pulmonary artery conduit replacement procedure can be achieved with a low surgical morbidity or mortality, not influenced by the type of conduit that is replaced. Therefore, the choice between homograft or Contegra® for right ventricle to pulmonary artery reconstruction should not be influenced by the future surgical risk during the replacement procedure.

**Trial registration:**

NCT03048071. Registered 9 February 2017 (retrospectively registered).

## Background

The use of a valved conduit between the right ventricle and the pulmonary artery (RV-PA) for right ventricular outflow tract (RVOT) reconstruction in congenital heart diseases was introduced by Ross and Somerville in 1966 [1]. Since then, the practice has gained in both popularity and use. Currently, cryopreserved homografts, and particularly pulmonary homografts (PH), are considered first-choice conduits. Regrettably, there is both a lack of availability [2] (especially in the smallest sizes more suitable for newborns) and an issue of early degeneration, particularly in younger patients [3, 4]. These obstacles prompted the development of alternative options. One such solution is Contegra®, a bovine jugular vein xenograft, which was introduced in 1999 by Medtronic® Inc. (Minneapolis, MN, USA). Contegra® showed excellent results in early, mid, and long term studies [5–9], so that some centers started recommending it as the preferred conduit for RVOT reconstruction [6]. This optimism has been tempered by several negative reports [10–12] revealing development of distal stenosis, aneurysms of the conduit, and a greater risk of endocarditis. Some teams, such as Göber et al. did not recommend its routine use [12].

Furthermore, whether homografts or Contegra®, RV-PA conduits lack growth potential. This, combined with their degeneration and/or calcification over time, poses a serious problem when used in young patients with a great growing potential, therefore requiring multiple RV-PA conduit replacements [13]. Despite the issues, and the number of years the surgery has existed, the RV-PA conduit replacement procedure’s results remain unknown, and poorly studied in the field of comparison between homografts and Contegra®. The purpose of this study is therefore to compare the surgical morbidity and mortality of Contegra® and homograft replacement in a pediatric population.

## Methods

### Patients

This study focuses on the RV-PA conduit replacement procedures, considering all the operations between January 1999 and December 2016 at the “Hôpital Universitaire des Enfants Reine Fabiola”. A total of 87 procedures were conducted on 82 children (49 boys, 33 girls). The procedures were divided into two groups: Contegra® or homograft replacement. Some were performed multiple times on the same patient (*n* = 5). Among these, three underwent one Contegra® replacement followed by one homograft replacement, one underwent two homograft replacements, and one underwent a homograft replacement followed by a Contegra® replacement. A total of 60 Contegra® and 27 homografts replacements (four aortic homografts and twenty three PH) were performed.

### Statistical methods

Statistical analyses were performed with the R software (R Core Team, 2016) version 3.2.2. We performed 15 multiple imputations, using the “Multivariate Imputation by Chained Equations” (MICE) R package [14] to reduce bias introduced by missing values. A propensity score was then used to match our two groups (Contegra® (*n* = 60) and homograft (*n* = 27) replacements) on a set of covariates (age to replace the conduit, duration of implantation, weight, gender, extra-anatomic position of the conduct vs anatomic position vs post-Ross procedure and concomitant procedures), allowing the mean and standard deviations as well as proportions to closer between the two studied groups. We then performed 15 linear or logistic regression, one on each imputed dataset and averaged the 15 *p*-values [15]. We used the “Covariate Balancing Propensity Score” (CBPS) R package, that uses covariates balancing and requesting an exact match to calculate the propensity score [16]. An Absolute Standardized Difference (ASD) of less than 10-15% was considered to support the balance between the groups. Finally, we used the Survey R package to perform logistic regressions for binary outcome variables and linear regressions for continuous outcomes. It considers the treatment group effect, the weight resulting from the matching and variables present in the propensity score. This provides a doubly-robust estimator which corrects the last remaining possible imbalance between the covariates and produces an unbiased treatment effect [17].

### Data collection and definitions

The data was retrieved through a retrospective review of the 82 medical records to include preoperative data, medical history, operative reports, anesthesia reports, intensive care unit (ICU) stay reports, pathology reports, computed tomography scans and echocardiography protocols. The two groups were then assessed based on covariates such as: age of explantation, duration of the implantation, weight, gender, the position of the conduit, and the existence of a concomitant procedure during the conduit replacement. In this study, the anatomical position of the conduit concerns conduits implanted for tetralogy of Fallot and pulmonary valve agenesis. The extra-anatomic position concerns truncus arteriosus, double atrio-ventricular discordance, double outlet right ventricle and all the conduits implanted during a Rastelli procedure [18].

For the preoperative results, we analysed anesthesia, surgery, cardiopulmonary bypass and aortic clamping durations, the existence of an operative complication and the fluid input and blood loss. The study defined an operative complication as an unusual and potentially harmful event occurring during the surgical procedure which was reported in the operative report. Those results include re-entry injuries that are defined as injury to cardiac or vascular structures during the sternotomy or during the dissection before cannulation for cardiopulmonary bypass [19]. The fluids input was divided in two categories: blood product input, including red blood cell concentrates, plasma, platelets and other fluid input, including crystalloids, colloids and albumin solutions.

The post-operative data, analyzed the Pediatric Risk of Mortality (PRISM) score which is a physiologically based score that can compute expected mortality risk and expected morbidity risk [20].

### Operating details

The same team of surgeons performed all the procedures through median sternotomy followed by the dissection of the cardio sternal and cardio pericardial adherences. The procedures were achieved using cardiopulmonary bypass, with or without hypothermia. Aortic clamping was performed in 17 procedures. All the calcifications and residual tissue were removed when possible. In most cases the conduit was entirely extracted. In several cases, in order to preserve the left main coronary artery, the posterior part of the conduit was left on site. In some cases, the prevalvular part of the conduit was left on site and the new conduit’s proximal anastomosis was performed on it. The aorta and the RV-PA conduct were protected using a Gore-Tex® membrane (especially in extra-anatomic position) or rarely the autologous pericardium. It allows an isolation of the sternum, creating a cleavage plane, which likely makes the re-sternotomy safer.

## Results

### Propensity score

Table 1 presents the propensity scores obtained. The left part of the table (before matching) reports the means and standard deviations for continuous data, shows proportions for discrete data, and the standardized difference in the two groups (Contegra® and homograft replacement). In almost all cases, the two groups have different means/proportions for the variables before matching and therefore cannot be compared as such. The right part of the table presents the results after matching. After matching means standard deviations, and proportions are comparable. The standardized difference values indicate that the two groups have similar means/proportions for the different variables after matching. Based on this matching table, the Contegra® and homograft groups are considered to be similar on covariates chosen for the propensity score.

**Table 1:** Demographics, conduit positions, and overall propensity scores

	Before Matching			After Matching		
Variables	Contegra® (*n* = 60)	Homograft (*n* = 27)	ASD	Contegra® (*n* = 60)	Homograft (*n* = 27)	ASD
Age at explantation (months)	86.43 ± 53.20	114.74 ± 59.69	50.07%	95.55 ± 56.88	95.55 ± 61.09	0.00%
Implantation duration of the 1st conduit (months)	68.18 ± 49.84	102.81 ± 54.69	66.19%	80.94 ± 57.06	80.94 ± 53.46	0.00%
Weight (kg)	22.83 ± 14.58	31.02 ± 18.69	48.82%	24.88 ± 14.81	24.88 ± 18.43	0.00%
Gender (female)	36.67%	51.85%	30.94%	45.55%	45.55%	0.00%
Extra-anatomic position	63.33%	44.44%	38.59%	59.46%	59.46%	0.00%
Anatomic position (excepted Ross)	23.33%	51.85%	61.62%	30.38%	30.38%	0.00%
Ross situation	13.34%	3.70%	35.03%	10.16%	10.16%	0.00%
Concomitant procedures	40.00%	29.63%	21.90%	34.48%	34.48%	0.00%

ASD: absolute standardized difference; kg: kilograms

In the Contegra® group, the degradation mode of the conduit was a pure stenosis in 27 patients, pure regurgitation in 5 and both in 27. The presence of endocarditis was demonstrated in 7 patients with combined stenosis and regurgitation, and in 3 patients with pure stenosis a thrombus in the conduct was objectified. Four patients reveal the presence of a distal peel (3 in pure stenosis and one both). In the homograft group, there were pure stenosis in 14, pure regurgitation in 2 and both in 11. The 4 aortic homografts have been degraded according to the mode of pure stenosis.

### Peroperative data: Table 2 presents the peroperative data

**Table 2:** Per operative data

	Contegra® (*n* = 60)	Homograft (*n* = 27)	Adjusted *p*-value
Anesthesia duration (min)	345.95 ± 79.05	356.22 ± 92.83	0.4635
Surgery duration (min)	221.61 ± 71.04	215.56 ± 41.13	0.5976
CBP duration (min)	99.40 ± 40.39	97.93 ± 36.08	0.908
Aortic clamping duration (min)	11.99 ± 28.38	11.44 ± 23.68	0.7364
Operative complication	13.47%	15.36%	0.758
Blood losses (ml)	581.76 ± 216.36	551.46 ± 185.80	0.4641
Blood products inputs (ml)	351.81 ± 217.93	428.03 ± 197.08	0.1533
Other fluids inputs (ml)	810.74 ± 407.05	901.94 ± 412.71	0.2599

A Bonferroni correction must be applied for multiple comparison purposes and the *p*-value (0.05) must be divided by the number of comparisons (*n* = 8), that is, 0.05/8 = 0.00625, to get the final p-value on which we can draw conclusion. Thereby, any p-value below 0.00625 are considered as significant. CBP: cardiopulmonary bypass; min: minute; ml: milliliters.

No statistically significant difference was observed between our two groups for the considered operative covariates that were anesthesia, surgery, cardiopulmonary bypass and aortic clamping durations (adjusted *p* value = 0.00625). In the two groups, the main cause of replacement was a stenosis of the conduit associated with a valvar insufficiency. Calcifications were observed in 48% (*n* = 29) of the explanted Contegra® and in 52% (*n* = 14) of the explanted homografts. During Contegra® or homograft replacements, an operative complication occurred in 13.47% and 15.36% respectively (adjusted *p*-value = 0.00625). The most common complication in the series were re-entry injuries (2 Contegra® and 2 homograft groups), three of which required an emergency conversion to a femoro-femoral cardiopulmonary bypass. Two of these re-entry injuries resulted in a hemothorax requiring further operation to drain the blood and remove the clots. Two air embolisms were also reported in the Contegra® group. We suspected a residual intracardiac shunts that were not preoperatively identified. A postoperative CT-scan determined that both patients required treatment by hyperbaric chamber. Two per-operative ventricular fibrillation episodes (*n* = 2 Contegra®) were observed requiring three external shocks and the administration of xylocaine for one, and an internal shock for the other. One allergic reaction to protamine (homograft group) was also reported, causing blood pressure elevation and difficult ventilation. One bilateral pneumothorax (Contegra® group) of unknown origin that required per-operative placement of two pleural drains, and one hemorrhage requiring revision for bleeding and transfusion of plasma and platelets were reported.

With respect to blood loss, blood input or other fluid input, there was no statistically significant difference (adjusted *p*-value = 0.00625).

### Postoperative outcomes

A Bonferroni correction must be applied for multiple comparison purposes and the p-value (0.05) must be divided by the number of comparisons (*n* = 6), that is, 0.05/6 = 0.0083, to get the final *p*-value on which we can draw conclusion. PRISM: Pediatric Risk of Mortality; ICU: intensive care unit; PO: postoperative; d: day; h: hours.

At admission to the ICU, the PRISM score is calculated for all patients in our institution after cardiac surgery. It was respectively 6.12 for Contegra® and 4.46 for homograft replacements, but not statistically significant (adjusted p-value = 0.0083).

To analyze postoperative morbidity, we considered the day of extubation, the day of withdrawal of inotropic drugs, the length of the ICU stay and the length of hospital stay. As mentioned in Table 3, there was no statistically significant difference between the two types of conduit. The lengths of the ICU stay and of hospitalization were substantially influenced in the Contegra® group by two patients whose hospitalizations had to be prolonged for other reasons than the RVOT reconstruction reoperation. The median duration of the ICU stay and hospitalization were respectively 4 days and 13.5 days for Contegra® and 3 days and 10 days for homografts.

**Table 3:** Postoperative evolution

	Contegra® (*n* = 60)	Homograft (*n* = 27)	Adjusted *p*-value
PRISM Score	6.12 ± 5.06	4.46 ± 2.64	0.0453
Inotropic drugs withdrawal (PO d)	1.59 ± 5.21	1.16 ± 1.41	0.4219
Extubation day (PO h)	24.48 ± 62.16	12.72 ± 16.08	0.1484
ICU stay (d)	13.97 ± 77.94	4.14 ± 1.88	0.2011
Hospital stay (d)	28.90 ± 103.04	13.85 ± 11.13	0.1579
Death	2.37%	0.00%	0.301

No death was observed during the post-operative course in the Homograft replacement group. Two deaths (2.37%) occurred in the Contegra® group. The first death occurred in a 19-month-old boy because of a massive air embolism of unknown origin occurred during cardiopulmonary bypass. The patient suffered from pulmonary atresia with ventricular septal defect and major aortopulmonary collateral arteries, palliated with a Blalock-Taussig shunt at one day of life. His Contegra® was implanted at the age of seven months. The Contegra® had to be replaced during an emergency procedure due to its acute degradation, most likely because of the protrusion of a peel on its distal part responsible for a dilation of the right cavities of the heart. The patient was conducted to a hyperbaric chamber right after the surgery. The MRI performed after 18 h showed a supratentorial cerebral edema with extensive cortical necrosis, injury of the basal ganglia, the mid brain and the pons, and the quasi absence of cerebral flow. He died one day after the surgery. The second death was a six-and-a-half-year-old girl. She suffered from a transposition of the great arteries with ventricular septal defect and pulmonary stenosis. She had first been palliated at the age of four months by a left modified Blalock-Taussig shunt, and at the age of 12 months by a right modified Blalock-Taussig shunt. At the age of six, she underwent a Rastelli procedure with the implantation of a 18 mm Contegra® conduit. During the weeks before the replacement procedure, she developed pneumonia and endocarditis on her Contegra®, was admitted to the ICU and treated by several rounds of antibiotics. The procedure was marked by an episode of ventricular fibrillation reduced by an internal shock of 15 joules. It also required the closure of a Swiss-cheese septum which required an aortic clamping time of 84 min and a cardiopulmonary bypass time of 201 min. The analysis of the removed Contegra® showed thrombosis of the conduit. Her post-operative course was marked by hospital-acquired pneumonia and by the development of a biventricular cardiac failure that was beyond any medical or surgical treatment. She died of a biventricular cardiac failure 31 days after the surgery was performed, 44 days after her admission to the ICU. Notwithstanding, this difference is not statistically significant (adjusted *p*-value = 0.301).

## Discussion

In many CHD cases, both RVOT reconstruction and the Ross procedure require the implantation of a RV-PA valved conduit. Despite advancements, it is likely that in most patients, this conduit will need to be replaced during their childhood, especially since early replacement of the conduit could prevent irreversible structural myocardial changes [21]. The feasibility of such a procedure is important to consider when implanting RV-PA conduits. Conduits like Carpentier-Edwards conduit, a porcine-valved Dacron conduit, are considered easy and safe to replaced [22] because they are easily pealed from the surrounding tissue. For this reason, some teams still use it in RVOT reconstruction.

Most surgeons consider PH as the “gold standard” for RVOT reconstruction, but it is not the “perfect conduit”. Despite providing good hemodynamic results without the need for anticoagulation [23], the increasing demand over the last 30 years and limited availability could lead to a shortage [2]. Furthermore, worries were raised concerning its durability, especially in younger patients [24, 25]. Wells et al. found a failure rate of PH of about 40% after 10 years [24], which concurrence with Boethig et al.’s findings of a 46% degeneration rate in patients older than 10 years of age, and a degeneration rate of 65% in younger patients after merely five years [26]. Several modes of conduit failure have been described, including shrinkage of the conduit, early calcifications, RVOT aneurysm, conduit valve insufficiency [24, 25, 27, 28], and outgrowth, although the latter occurs less than expected [3, 24]. Risks factors for homograft failure have been extensively studied. They include younger aged recipients, smaller homograft diameter, the use of aortic homograft in older patients, low weight at implantation, truncus arteriosus and an extra-anatomic position [3, 24, 28, 29]. Moreover, in 2010, Christenson et al. showed that the performances of non ABO-matched homografts were significantly worse than ABO-matched homografts, especially for younger children [23]. In Belgium, the homografts are never ABO-matched. Studies have shown that replaced homograft performs as well as the first homograft [13, 30]. Technical considerations may influence the surgeon’s preference for a conduit. PH implantation requires additional prosthetic or pericardial patches to connect to the right ventricle. All these considerations may render the PH imperfect for newborns [31].

Given the aforementioned considerations, the Contegra® conduit was introduced in 1999 for RVOT reconstruction. In Belgium, Contegra® has been available since May 2000 and since then, unsupported Contegra® conduits have been used. Contegra® is a bovine jugular vein conduit with a native triple-leaflet valve stored in diluted and buffered glutaraldehyde. This retains leaflets and conduit wall compliance and likely renders Contegra® non-antigenic [32]. This differs from cryopreservation which does not eliminate the antigenic expression in homografts [33]. Unlike PH, Contegra® is readily available in sizes ranging from 12 mm to 22 mm. Moreover, studies are optimistic, showing good hemodynamic results [34]. Some authors even consider Contegra® as a valid alternative to homografts [5, 6, 8, 23, 34–37], even in newborns [31]. In 2011, Brown et al. described a freedom from explantation for Contegra® at both five and ten years of 97% and 90% respectively, versus 81% and 69% for PHs [35]. It has been observed that degradation is characterized by less calcification [6, 7, 34]. As for technical considerations, Contegra® because of the abundance of tissue on both sides of the valve, does not need additional material for proximal hoods and distal pasties. Suturing is simple, the material is resistant to suture tear and very flexible, therefore, making its surgical handling easy. Concerns were raised due to a specific complication with Contegra®: stenosis at the distal anastomosis which causes proximal conduit dilation and the formation of an aneurysm in small sized conduits [10–12, 38], so that some authors discouraged its use [10]. Since the modification of the operative technique as proposed by Corno et al. in 2008, this complication has become rarer [8, 38]. Few cases of pseudo aneurysm of the right ventricle after Contegra® implantation have been reported [39]. Such a complication occurred once in the present study. The seven-and-a-half-year-old patient underwent a Rastelli procedure six years earlier with the implantation of a 18 mm Contegra®, to correct a transposition of the great arteries with ventricular septal defect and pulmonary stenosis. The pre-operative transesophageal echocardiography showed a dilation of the right ventricle and a subaortic membrane. The false aneurysm was located between the ventriculotomy and the Contegra® which was entirely disconnected from the ventricle. The Contegra® was removed and replaced by an 18 mm Contegra®. More recently, Ugaki et al. reported a further complication: endocarditis, which occurred in 9% of the Contegra® implanted, 10 times more often than what is observed with homografts, particularly in children older than three years [40]. Another source of concern is the risk of thrombus formation as reported by Tiete et al. [12, 41] and for which they recommend a prophylactic anticoagulation. All patients with a Contegra® received low molecular weight heparin during two months after the implantation in our center, which might explain the small incidence (2 cases; 3.3%) of Contegra® replacement due to thrombosis.

Low surgical mortality is reported and death rate is identical between homograft and Contegra® replacements, confirming other reports that proclaim it to be a safe procedure [13, 27, 42]. It suggests that the type of replaced conduit does not influence the perioperative mortality. The overall operative complication rate was approximately 14%, even though the patients underwent multiple sternotomies. This rate was not influenced by the type of conduit in place during the redo procedure and this result corroborates with other reports on the fact that, if approached meticulously in specialized centers, repeat sternotomy can be performed with a minor subsequent morbidity and mortality [43, 44]. Allografts and xenografts differ in the host immunogenic response, and when implanting a xenograft, one could expect a greater inflammatory reaction. For Wojtalik et al. the three reasons for potential immunogenicity and antigenicity of Contegra® are: a big surface of contact with host tissues, cellular remnants (whole endothelial layer) within the implant and heterogeneous graft fixation by glutaraldehyde [45]. This can cause more adherences (therefore affecting the surgery duration), and ultimately more rejections than homografts. No difference was observed in terms of duration, bleeding or transfusion between the two groups. From an operative point of view, calcifications are observed in the two groups, but in the Contegra® group, they tend to be less extensive than the ones observed in the explanted homografts group. Both groups present adherences. Their dissection and the explantation of the conduit were easier in the Contegra® group thanks to a cleavage plane well defined by the tunica externa of the bovine jugular vein, which once removed, allows an easy and safe dissection. Finally, no difference in post-operative morbidity was observed between the two groups, suggesting that the perioperative course is not affected by the type of the explanted conduit.

## Conclusion

In conclusion, the “perfect RV-PA conduit” is yet to be found, and both Contegra® or homografts used in pediatric RVOT reconstruction will require explantation and replacement. Multiple RVOT reconstruction with RV-PA conduit replacement can be performed safely with a low surgical morbidity or mortality. We showed that the type of conduit does not influence these results. Therefore, the choice of conduit for RVOT reconstruction should not be influenced by the future surgical risk during the replacement procedure.

### Limitation of the study

Limitations of the present report are inherent with the retrospective nature of the study and the small number of patients.

## Abbreviations

BJV: Bovine jugular vein
CBPS: Covariate balancing propensity score
CHD: Congenital heart diseases
ICU: Intensive care unit
MICE: Multivariate imputation by chained eqs.
PH: Pulmonary homograft
PRISM: Pediatric risk of mortality
RVOT: Right ventricular outflow tract
RV-PA: Right ventricle to pulmonary artery
